# Recommendations and optimal approaches to robotic-assisted partial nephrectomy: A consensus of Brazilian experts

**DOI:** 10.3389/fruro.2023.1119494

**Published:** 2023-02-03

**Authors:** Eliney Ferreira Faria, Marcio Covas Moschovas, Carlos Vaz, Alexandre Pompeo, Alexandre Santos, Alexandre Stievano, Andre Berger, Arie Carneiro, Aurus Dourado, Jose Roberto Colombo, Carlo Passerotti, Cassio Andreoni, Clovis Fraga, Giuliano Guglielmetti, Gustavo Lemos, Gustavo Guimarães, Lucas Nogueira, Marcos Rocha, Pablo Melo, Paulo Arantes, Pedro Romanelli, Rafael Tourinho, Ricardo Nishimoto, Roberto Machado, Rodolfo Reis, Rodrigo Frota, Romulo Guida, Victor Dubeux, Rodrigo Gualberto, Marcos Tobias-Machado

**Affiliations:** ^1^ Urology Department, Hospital Felício Rocho, Belo, Horizonte, Brazil; ^2^ Urology Department, Advent Health, Orlando, FL, United States; ^3^ Urology Department, Beneficência Portuguesa de São Paulo, São Paulo, Brazil; ^4^ Urology Department, Hospital Beneficiencia Portuguesa de Rio Preto, Rio Preto, Brazil; ^5^ Urology Department, Hospital Santa Marcelina, São Paulo, Brazil; ^6^ Urology Department, Hospital Moinhos de Vento, Porto Alegre, Brazil; ^7^ Urology Department, Albert Einstein Israelite Hospital, São Paulo, Brazil; ^8^ Urology Department, Hospital São Marcos, Terezina, Brazil; ^9^ Urology Department, Hospital Alemão Oswaldo Cruz, São Paulo, Brazil; ^10^ Urology Department, Real Hospital Português, Recife, Brazil; ^11^ Urology Department, Hospital das Clinicas FMUSP, São Paulo, Brazil; ^12^ Urology Department, Hospital das Cínicas UFMG, Belo Horizonte, Minas Gerais, Brazil; ^13^ Urology Department, Hospital Geral de Fortaleza, Fortaleza, Brazil; ^14^ Urology Department, Hospital Cardiopulmonar, Salvador, Brazil; ^15^ Urology Department, Hospital Madre Teresa, Belo Horizonte, Brazil; ^16^ Urology Department, Hospital do Amor, Barretos, Brazil; ^17^ Urology Department, Hospital Quinta Dor, Rio de Janeiro, Brazil; ^18^ Urology Department, Hospital Universitário Pedro Ernesto, Rio de Janeiro, Brazil; ^19^ Urology Department, Instituto do Cancer Arnaldo Vieira de Carvalho, São Paulo, Brazil

**Keywords:** kidney, kidney cancer, robotic surgery, partial nephrectomy, nephrectomy

## Abstract

**Objective:**

Robotic-assisted partial nephrectomy (RAPN) is established as the gold standard approach to treating small renal masses. However, numerous technical challenges and concepts related to this approach are still under discussion and are not consensus among surgeons from different centers. We performed an online questionnaire with multiple topics about RAPN and selected high-volume surgeons from referral centers in Brazil to achieve a consensus.

**Methods:**

We implemented an online consensus of 29 experts selected based on surgical expertise and competence in analyzing the published literature. Based on the collected literature and current Guidelines (NCCN, AUA, and EAU) we created a questionnaire with 131 questions and administered it to all participants. The statements and the Delphi technique design were combined in a single round of questions. The answers were reviewed, 70% of concordance was considered a consensus, and a final manuscript with recommendations was developed.

**Results:**

We divided our results into 25 subtopics that included all questions and discussions of the questionnaire, including preoperative settings, surgical technique, pathological analysis, technology use, and challenging cases. Some areas had limited data in the literature, and these potential limitations were addressed and discussed on each topic.

**Conclusion:**

RAPN is the standard surgical treatment for renal masses in the centers of robotic surgery. Among the important topics of this study, we recommend always performing the first RAPN cases with proctors’ assistance, conducting preoperative planning using good-quality imaging exams, minimizing the amount of renal parenchyma removed, and achieving appropriate hemostatic suture while reducing renal parenchyma ischemia.

## 1 Introduction

Robotic-assisted partial nephrectomy (RAPN) is the established gold standard treatment of small renal masses in several centers worldwide. This technique has been standardized and had rapid expansion with feasible and reproducible outcomes even in complex cases (1). However, several technical issues related to RAPN remain unclear in the literature and among surgeons worldwide. In this scenario, we conducted a consensus of experts based on the surgeon’s experience, literature review, and current guidelines recommendations (NCCN, AUA, EAU) on RAPN.

## 2 Methods

First, we selected 29 experts based on their robotic surgery experience, number of surgeries performed, regular access to robotic platforms, and competence in critically were selected to participate. We considered expert surgeons with at least 100 robotic-assisted radical prostatectomies and at least 30 partial nephrectomies.

Second, we established the research topic as “robotic-assisted partial nephrectomy” and all subjects involved with this surgery, such as preoperative images, operative routine, technique, and challenges. We considered only procedures performed with the Da Vinci robotic platform.

Third, 23 experts reviewed the literature (using the PRISMA method) and current Guidelines (NCCN, AUA, and EAU) from March 2021 to September 2021 and prepared a multiple-choice questionnaire (4 and 6 alternatives for each question). Fourth, five experts (EF, MCM, CV, PM, MTM) selected the most appropriate questions to add to the final manuscript. Fifth, we administered an online questionnaire to all participants, analyzed all responses, and selected the consensus answers. Finally, three experts (EF, MCM, MTM) prepared the final manuscript with the approval of all participants. The study design and process are illustrated in Flowchart 1.

### 2.1 Evidence synthesis

We reviewed all RAPN publications (written in English) in the PubMed^®^ (Medline) database until September 2021. The statements and the Delphi technique design (2) were used in a single round, in which 131 multiple-choice questions were sent to all participants by email. All data were collected and stored in a dataset using REDCap^®^ (Research Electronic Data Capture). The reviewers worked on the final recommendations using consensus and non-consensus questions, selecting those most relevant for publication. The consensus was considered when 75% or more experts had the same opinion. The crucial aspects of our questionnaire are illustrated in the **Supplementary Table 1**.

## 3 Results

### 3.1 Bowel preparation and antimicrobial prophylaxis

The current literature does not show the advantages of RAPN regarding surgical time, hospitalization time, or peri-operative complications (3). Our consensus did not recommend routine bowel preparations for RAPN.

There was no consensus regarding prophylactic antibiotics for RAPN. However, patients with advanced age, anatomic urinary tract anomalies or calculi, nutritional deficiency, immunodeficiency, colonization, or exogenous implants may all benefit from directed antimicrobial prophylaxis (4).

### 3.2 Preoperative imaging

It is recommended that a preoperative contrast-enhanced computed tomography (CT) or magnetic resonance imaging (MRI) of the abdomen should be performed (5). Information related to the function and morphology of both kidneys, the extent of the tumor, venous involvement, lymph node involvement, degree of local invasion, and number and position of renal arteries and veins is essential for surgical planning (6, 7). For the evaluation of complex cystic lesions, MRI has the advantage of possessing higher sensitivity and specificity in detecting cystic tumors (8). There was unanimity in recommending CT and/or MRI for the preoperative evaluation. According to our experts, tumor size and location are the two most relevant factors for RAPN.

### 3.3 Three-dimensional reconstructions, 3D printing, and augmented reality

Several software programs can perform 3D reconstructions and, more recently, 3D mold impressions. Furthermore, software based on augmented reality is available to assist the RAPN. Preliminary studies have reported a change in surgical strategy, increased surgeon subjective confidence, and a better understanding of renal anatomy using these technologies (9–12).

In our panel, 62.5% of the experts recommended using 3D reconstruction for tumors with high nephrometry scores. However, a cutoff point was not determined. Seventy-eight percent of the experts indicated that augmented reality could be a valuable tool in managing hilar and endophytic lesions.

### 3.4 Intraoperative ultrasound

The intraoperative US was recommended for completely endophytic, multiple, and complex tumors by 100%, 87.5%, and 21% of the experts, respectively. The literature also recommends using the intraoperative US in multiple, unilateral, bilateral, and endophytic lesions (13–15).

### 3.5 Nephrometric scores

Several scores have been used to classify tumor complexity based on renal anatomy,such as ABC (16), PADUA (17), RENAL (18), mathematical scores (19), and scores to predict the degree of adhesion of peri-renal fat (20). Although these scores can predict the surgical time, length of stay, ischemia time, blood loss, complications, and the likelihood of conversion (21), evidence that they are superior to the surgeon’s subjective assessment is lacking. Only 50% of experts believed that the nephrometry scores should be routinely used, and a preference for specific Nephrometric scores was not expressed. A high nephrometry score is not an impeditive factor for performing RAPN.

### 3.6 Peri-renal adhesive fat

The experts were unanimous in considering PAF as a factor directly associated with the complexity of the RAPN. Although most experts did not use nomograms to assess PAF, 75% used clinical data such as perinephric adipose tissue thickness, stretch marks, male sex, high body mass index (BMI), hypertension, and diabetes mellitus as the predictors of PAF. Our experts understood that the impact of this condition on the oncological outcomes of RAPN was controversial.

PAF has been associated with higher blood loss, longer surgical time, longer warm ischemia time (WIT), prolonged hospital stay, and higher complication rates (20, 22).

### 3.7 Training and learning curve

One of the questions to be addressed while analyzing LC in robotic surgery is whether the surgeon’s previous experience with laparoscopic partial nephrectomy (LPN) accelerates the LC of RAPN to the point that LPN is a prerequisite for RAPN (23). Faria et al. (24) showed that the robot could reduce WIT even for surgeons experienced in laparoscopy. In our consensus, 84% of the experts believed that experience in laparoscopy accelerated the LC of RAPN. However, it is not an essential condition for RAPN training. Among the experts, 84% believed that the surgeon in training should start with less complicated cases, and 96% of the experts believe that it is mandatory to perform initial cases under supervision.

### 3.8 Trocar placement and robotic instruments

There was no consensus on trocar positioning in RAPN. However, 39% of the experts considered that the position of the trocar varied according to the patient’s body habitus, size and location of the tumor, and type of robotic platform used.

Regarding the robotic forceps instrument, the only disagreement among the experts was the use of bipolar fenestrated forceps (50%) or bipolar Maryland (50%). The other forceps routinely used by the urologists of the consensus group were Prograsp, monopolar scissors, and needle holders. According to our experts, the number of arms used could vary according to the robotic platform (SI *vs*. Xi) and patient habitus. Of our experts, 54.2% used one auxiliary portal, and 37.5% used two portals.

### 3.9 Transperitoneal *vs* retroperitoneal approach

Pavan et al. (25) conducted a systematic review to evaluate the perioperative outcomes of transperitoneal and retroperitoneal approaches for RAPN in 886 and 513 patients, respectively. There were no differences in overall complications, postoperative complications, WIT, and positive margins. The experts used the transperitoneal approach for most tumors in the RAPN, leaving the retroperitoneal approach for selected cases (high probability of abdominal adhesions from previous surgeries or inflammatory diseases and superobese patients).

### 3.10 Positive surgical margins and local recurrence

Local recurrence after partial nephrectomy ranged from 1.4% to 10% of patients. The main risk factors were the advanced tumor stage and high tumor grade (26, 27). Positive surgical margins may have prognostic implications only in high-grade or advanced-stage disease and may lead to poor recurrence-free and metastasis-free survival. Total nephrectomy should be considered in cases with extensive and high-grade positive margins (26, 28).

The consensus recommended that in the case of PSM in the surgical specimen, the patient should be observed, whereas if the margin was extensive, the patient should be counseled regarding the high risk of local recurrence. At the same time, radical nephrectomy or complementary partial nephrectomy should be proposed if there is evidence of radiological or histological recurrence.

### 3.11 Follow-up

There is no consensus regarding the strategies or optimal duration of patient follow-up after local treatment of renal tumors.

### 3.12 Simple Tumor Enucleation and classic partial nephrectomy

Simple Tumor Enucleation (STE) is an alternative to Classical Partial Nephrectomy (CPR) (29). CPR is mainly associated with some degree of functional decline related to the excised healthy parenchyma and to devascularization that may occur during the reconstructive phase of the procedure. STE is associated with a shorter operative time and lower blood loss. Some authors argue that routine reconstruction of the parenchyma may not be necessary after tumor resection if there is minimal bleeding, unlike CPR (30, 31). In a classic European multicenter study, STE was associated with cancer-specific survival outcomes equivalent to CPR (29). The decision for enucleation should be based on the tumor size and location and the patient’s preoperative renal function.

Even in cases of tumor enucleation, routine renal repair was recommended by 78.3% of the experts in the consensus. Regarding the preservation of renal function, 83% of the experts believed that the amount of preserved renal parenchyma was the most crucial factor in preserving function. Whereas 41.7% believed that the WIT was equally relevant, only 16.7% of the experts considered WIT to be the main factor in determining renal function preservation.

### 3.13 Margin thickness

The average thickness of the safety margin around the tumor varies from 2.5 mm to 5 mm. Some authors have demonstrated that negative surgical margins can result in an ideal thickness of 5 mm (32). The EAU guidelines recommend the presence of a minimal surgical margin of healthy renal parenchyma around the resected tumor to reduce the risk of local recurrence or progression. However, the exact minimum thickness of the parenchyma to be resected was not specified (33). This variability in safety margin thickness may be influenced by various anatomical and topographical features of the tumor. Our experts agreed that there was no consensus in the literature regarding the thickness of healthy tissue that should be excised along with the tumor to ensure a negative margin.

### 3.14 Tumor bed biopsy

Boris and collaborators recommended biopsy only as an option to be considered by the surgeons’ preferences (15). Hence, the consensus did not recommend tumor bed biopsy to evaluate residual disease.

### 3.15 Technical details of partial nephrectomy in T2, T3, and Ttumors

The current literature still lacks well-designed studies describing the role of RAPN for T2, T3, or T4 renal tumors. Available reports are controversial and based on retrospective studies with an inherent risk of bias. In addition, RAPN tumors with venous thrombus (T3) or those that are locally advanced (T4) are challenging with a significant risk of complication. Thus, the best surgical approach is still unknown. The consensus recommended that RAPN should be performed for T2, T3, and in selected cases of T4. However, it is very dependent on the surgeon’s experience.

### 3.16 Renal hilum clamping

Greco and colleagues analyzed 156 studies of partial nephrectomy comparing different ischemia techniques and reported the results in a systematic review and meta-analysis. They found that zero ischemia techniques were associated with greater positive surgical margins than other ischemia techniques due to the worst visualization of a tumor bed during RAPN (34). Therefore, this technique is not a consensus among the experts.

Several ischemic techniques have been described and used throughout the evolution of partial nephrectomy: cold ischemia, WIT with complete arterial clamping (renal artery trunk), selective ischemia, early de-clamping, and zero ischemia (no clamping) (35, 36).

According to the experts, the choice of technique depends on the surgical team’s experience, access used, and tumor characteristics (size, location, nephrometry) (34, 37, 38). Most perform individual arterial clamping, reserving venous clamping for complex hilar tumors, and performing the off-clamp technique in selected cases.

In the analysis performed by Greco et al. (34), there was no significant difference in intraoperative bleeding volume among the ischemia techniques used. Analysis of functional results did not point to the superiority of any technique over another, and the differences in the results were clinically insignificant.

### 3.17 Fluorescence by indocyanine green

Indocyanine green allows for real-time intraoperative identification of tumor versus normal renal tissue and assists in evaluating renal perfusion before and after clamping. In a prospective comparative study of 94 patients who underwent partial nephrectomy, ICG-NIRF (indocyanine green - near-infrared fluorescence) showed a decreased WIT (15 *vs*. 17 min, p = 0.01). There was no increase in the rate of PSM or complications in tumors with similar preoperative characteristics. In addition, patients in the ICG cohort underwent selective versus hilar clamping more frequently (39). There was no consensus on the use of ICG in our panel.

### 3.18 Reconstruction: medullary and cortical sutures

Several renal grafting techniques are described in the literature, varying according to the surgeon’s experience and the complexity of the tumor. Additionally, the approach adopted for tumor removal, resection, or enucleation may influence the type of reconstruction performed. There was no consensus on the ideal approach for renorrhaphy during partial nephrectomy (40–42). Moreover, there was not much data on the impact of renorrhaphy on long-term renal function (41).

When comparing continuous sutures versus individual stitches, the studies demonstrated the same results regarding complications and bleeding. However, ischemia time is shorter when a running suture is used. A continuous suture can still distribute tension better with less implanted material. Individual stitches are especially useful for high-tension sutures with significant and irregular defects. However, they are associated with prolonged renorrhaphy time and greater deposition of hemostasis materials (43). The experts agreed that early unclamping should be done whenever possible before a cortical suture is made to identify bleeding areas, reduce ischemia time, and aid in medullary suturing, which is also described in the literature (44).

In the case of hilar tumors, 79.2% of our experts stated that they performed certain tactical/technical modifications of the suture. In contrast, 91.7% believed that robotic technology was fundamental for reconstructions near the renal hilum.

### 3.19 Types of suture and needles

The ideal renorrhaphy should have tensile strength enough to perform hemostasis and avoid parenchymal ischemia.

A unidirectional barbed suture is equivalent in terms of tissue approximation to a conventional knotted suture (45). Several studies have been published comparing renorrhaphy with and without the barbed suture. The results showed safety and a significant reduction (about 27%) of WIT (46–51).

For sutures in the medullary layer, almost all experts recommended continuous sutures using 2.0 or 3.0 monofilament absorbable stitches that maintain tension for at least 14 days. Moreover, 50.4% of the experts preferred a 26 mm half circle/atraumatic needle. There was no consensus among the experts regarding using a barbed suture for the medullary layer: 50% used it routinely, 20.8% used it sometimes, and 29.2% never used it.

If there is an extensive opening of the collecting system or there are bleeding high-caliber vessels in the medullary layer, individual sutures of these vessels are recommended before proceeding to continuous suturing. According to our experts, 82.6% defined the medullary suture as the most important step for hemostasis. They also recommended focusing on the hemostasis of the medullary layer as a fundamental factor in avoiding the complication of postoperative bleeding. Moreover, it was recommended that during medullary renorraphy, needle passages should not be deep to prevent injuring the collecting system and to avoid unnecessary tissue ischemia.

There was no consensus about using continuous sutures or individual stitches regarding the cortical layer suture. Among the experts, 45.8% performed continuous sutures, and 50% used sutures with individual stitches. Of these, 20.8% used monofilament sutures, 50% used multifilament sutures, and 50% used scalloped sutures. In our consensus, 45.8% used 2.0 sutures, and 45.98% used sutures 0 or 1. Atraumatic needles of intermediate size (SH) or large size (CT) were recommended. Half of the experts used 2 cm CT needles, and 33% used 26 mm needles. Finally, in case of a vascular lesion (hilar vessels), the consensus recommended using non-absorbable sutures, 4-0 or 5-0 Prolene, and an atraumatic cardiovascular needle.

### 3.20 Use of polymer clips in renorrhaphy

Parenchymal reconstruction involving interrupted parenchymal sutures with polymer clips has been widely used in minimally invasive surgeries (52). This process reduces ischemia time and parenchymal laceration risk (41). All experts in the consensus used polymer clips to anchor the suture during renal repair, but 62.5% believed that the clips were not essential for the procedure.

### 3.21 Hemostatic agents

Among the experts, 83% did not believe that hemostatic agents were determinants in preventing hemorrhage. Regarding the use of these agents in clinical practice, 8.3% stated that they always used some hemostatic agent, 29.2% used it most of the time, 37.5% in selected cases, and 25% never used it. The need to use two hemostatic agents in selected cases was reported by 37.5% of the experts. There was no consensus regarding the definition or prohibition of the use of hemostatic agents during the opening of the collecting system and the quality of its suturing.

### 3.22 Multiple and bilateral renal tumors

Multiple renal masses make RAPN even more challenging, especially in cases of bilateral tumors (53, 54). Regarding bilateral tumors, the experts were divided (50%/50%) as to whether to start the approach from the side of higher or lower tumor complexity. In these cases, lesion enucleation was recommended by 96% of the experts.

Although some centers perform concomitant treatments, most centers prefer a two-stage approach with bilateral tumors because of the potential risks of complications and the immediate impact on renal function (55).

### 3.23 Hilar tumors

Radiological evaluation is essential to define the surgical technique. Simple enucleation is usually performed, and punctual medullary sutures are made to avoid compromising the main hilar vessels or collecting ducts. The Gil Vernet dissection is commonly employed, and not infrequently, tumor nourishing vessels are identified and ligated with polymer clips (Hem-o-lok ™) before hilar clamping. It was recommended that total clamping (artery and vein) should be considered in some hilar tumors because of the chances of increased blood loss.

### 3.24 Single kidney

In this consensus, 87.5% of the experts believed RAPN to be safe in patients with a single kidney, and 91.7% conveyed that robotic surgery results were compatible with those who underwent open surgery and better than those seen in LPN. There was also no increase in surgical conversion rates, and there were similar oncological results.

Perioperative RAPN outcomes were comparable with those who underwent open partial nephrectomy (OPN) in a retrospective analysis with 40 cases of RAPN and 85 cases of OPN. The surgical margin rates were 7.5% for RAPN and 8.5% for OPN, with no statistically significant difference. There were no statistical differences regarding operative complications, transfusion rates, positive surgical margins, and estimated glomerular filtration rate at one month. Patients in the RAPN group had shorter hospital stay (56).

### 3.25 Endophytic tumors

In cases of endophytic tumors, the panel suggested the use of intraoperative ultrasound. With intraoperative ultrasound, RAPN presents similar results as OPN in cases of endophytic and complex tumors (57).

## 4 Conclusion

Robotic-assisted partial nephrectomy is the standard surgical treatment for renal tumors in centers with access to robotic surgery. However, this procedure is not devoid of complications, and a consensus was not reached on some questions due to the lack of well-designed studies assessing these areas. Among the important topics of this study, we recommend always performing the first RAPN cases with proctors’ assistance, conducting preoperative planning using good-quality imaging exams, minimizing the amount of renal parenchyma removed, and achieving appropriate hemostatic suture while reducing renal parenchyma ischemia.

## Data availability statement

The original contributions presented in the study are included in the article/**Supplementary Material**. Further inquiries can be directed to the corresponding author.

## Ethics statement

Ethical review and approval was not required for the study of human participants in accordance with the local legislation and institutional requirements. Written informed consent from the participants was not required to participate in this study in accordance with the national legislation and the institutional requirements.

## Author contributions

All authors had substantial contribution to this work. EF and MM had equal contribution on this manuscript. All authors contributed to the article and approved the submitted version.

## Supplementary material

Supplementary Table 1 Consensus recommendations summary
