# Supplementary material for: Recommendations and optimal approaches to robotic-assisted partial nephrectomy: A consensus of Brazilian experts
Source: Front Urol. 2023 Feb 3;3:1119494. doi: 10.3389/fruro.2023.1119494 (PMC12327267; doi:10.3389/fruro.2023.1119494)
Supplement: Supplementary Table 1 — Consensus recommendations summary [file Table_1.docx]

**Supplementary table 1:** Consensus Recommendations Summary

| **Topic 1. Bowel preparation and Prophylactic antibiotics for RAPN** |
| --- |
| 1.1 Our consensus did not recommend routine bowel preparations for RAPN. |
| 1.3 There was no consensus regarding prophylactic antibiotics*.* |
| **Topic 2. Preoperative Imaging** |
| There was unanimity in recommending CT and/or MRI for the preoperative evaluation. According to our experts, tumor size and location are the two most relevant factors for RAPN. |
| **Topic 3. Three-dimensional (3D) reconstructions, 3D printing, and augmented reality** |
| 3.1 In our panel, 62.5% of the experts recommended the use of 3D reconstruction (no consensus). |
| 3.1 We had a consensus that augmented reality could be a valuable tool in the management of hilar and endophytic lesions. |
| **Topic 4. Intraoperative Ultrasound (US)** |
| Our experts recommended the intraoperative US use in RAPN. |
| **Topic 5.** **Nephrometric scores** |
| We had no consensus regarding the use of Nephrometric scores in RAPN. |
| **Topic 6. Peri-renal Adhesive Fat (PAR)** |
| The experts were unanimous in considering PAR as a factor directly associated with the complexity of the RAPN. |
| **Topic 7. Training and learning curve (LC)** |
| 7.1 Previous experience in laparoscopy: 84% of the experts believed that experience in laparoscopy accelerated the LC of RAPN. |
| 7.2 Initial RAPN cases: 96% of the experts believe that it is mandatory to perform initial cases under supervision. |
| **Topic 8. Trocar placement *and Robotic Instruments*** |
| 8.1 There was no consensus on trocar positioning in RAPN. |
| 8.2 Prograsp, monopolar scissors, and needle holders are the consensus among the experts. However, the use of bipolar fenestrated forceps (50%) or bipolar Maryland (50%) was not consensus. |
| **Topic 9.** **Transperitoneal vs. Retroperitoneal approach** |
| It is a consensus that the experts used the transperitoneal approach for most tumors. |
| **Topic 10. Positive surgical margins (PSM) and Local recurrence** |
| 10.1 The consensus recommended that in the case of a positive margin in the surgical specimen, the patient should be observed, whereas if the margin was extensive, the patient should be counseled regarding the high risk of local recurrence. |
| 10.2 It is consensus that radical nephrectomy or complementary partial nephrectomy should be proposed if there is evidence of radiological or histological recurrence. |
| **Topic 11.** **Follow-up** |
| There is no consensus regarding strategies or optimal duration of patient follow-up after local treatment of renal tumors. |
| **Topic 12. Simple Tumor Enucleation and classic partial nephrectomy** |
| It is consensus that routine renal repair should be performed, even in cases of tumor enucleation. Also, 83% of the experts believed that the amount of preserved renal parenchyma is the most crucial factor in preserving function. |
| **Topic 13. *Margin thickness*** |
| There was no consensus regarding the thickness of healthy tissue that should be excised along with the tumor to ensure a negative margin. |
| **Topic 14*. Tumor bed biopsy*** |
| The consensus did not recommend routine freezing biopsy of the tumor bed to evaluate residual disease. |
| **Topic 15. Technical details of partial nephrectomy in T2, T3, and T4 tumors** |
| The consensus recommended that RAPN should be performed for T2, T3, and in selected cases of T4. However, it is very dependent on the surgeon's experience. |
| **Topic 16.** **Renal hilum clamping** |
| Zero ischemia is not a consensus among the experts due to the worse visualization of the tumor bed with this technique. |
|  |
| **Topic 17.** **Fluorescence by indocyanine green (ICG)** |
| There was no consensus on the use of ICG in our panel. |
| **Topic 18. Reconstruction: medullary and cortical sutures** |
| 18.1 There was no consensus on the ideal approach for renorrhaphy during partial nephrectomy. |
| 19.2 The experts agreed that early unclipping should be done whenever possible before a cortical suture is made. |
| **Topic 19.** **Types of suture and needles** |
| 19.1 For sutures in the medullary layer, almost all experts recommended continuous sutures using 2.0 or 3.0 monofilament absorbable stitches. |
| 19.2 There was no consensus among the experts regarding using a barbed suture for the medullary layer. |
| 19.3 According to our experts, 82.6% defined the medullary suture as the most important step for hemostasis. They also recommended focusing on the hemostasis of the medullary layer as a fundamental factor in avoiding the complication of postoperative bleeding. |
| 19.4 Regarding the suture of the cortical layer, there was no consensus regarding the use of continuous suture or individual stitches. |
| 19.5 Regarding the cortical layer suture, there was no consensus about using continuous sutures or individual stitches. |
| 19.6 In case of a vascular lesion (hilar vessels), the consensus recommended using non-absorbable sutures, 4-0 or 5-0 Prolene, and an atraumatic cardiovascular needle. |
| **Topic 20.** **Use of polymer clips in renorrhaphy** |
| All experts in the consensus used polymer clips to anchor the suture during renal repair, but 62.5% believed that the clips were not essential for the procedure. |
| **Topic 21.** **Hemostatic agents** |
| It is not a consensus that hemostatic agents were determinants in preventing hemorrhage. |
| **Topic 22. *Multiple and bilateral renal tumors*** |
| Regarding bilateral tumors, the experts were divided (50%/50%) as to whether to |
| start the approach from the side of higher or lower tumor complexity. In these cases, lesion enucleation was recommended by 96% of the experts. |
| **Topic 23. Hilar Tumors** |
| It was recommended that total clamping (artery and vein) should be considered in some hilar tumors because of the chances of increased blood loss. |
| **Topic 24. Single Kidney** |
| It is consensus (87.5%) that RAPN is safe in patients with a single kidney. |
| **Topic 25.** **Endophytic Tumors** |
| In cases of endophytic tumors, the use of intraoperative ultrasound was suggested by experts. |
