# Supplementary material for: Recommendations and optimal approaches to robotic-assisted partial nephrectomy: A consensus of Brazilian experts
Source: Front Urol. 2023 Feb 3;3:1119494. doi: 10.3389/fruro.2023.1119494 (PMC12327267; doi:10.3389/fruro.2023.1119494)
Supplement: Supplementary file 2 [file DataSheet_1.pdf]

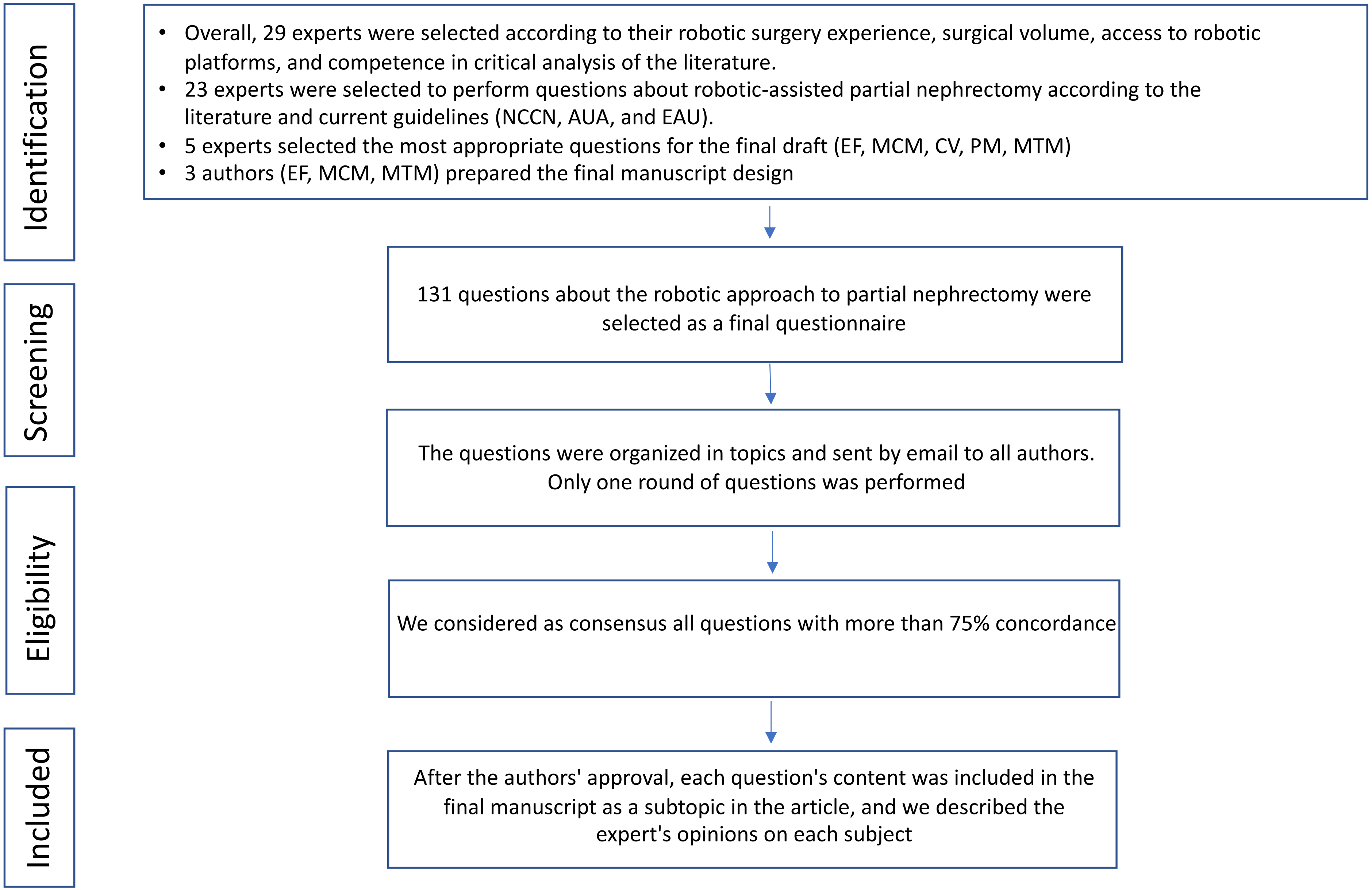
Identification
Overall, 29 experts were selected according to their robotic surgery experience, surgical volume, access to robotic platforms, and competence in critical analysis of the literature.
23 experts were selected to perform questions about robotic-assisted partial nephrectomy according to the literature and current guidelines (NCCN, AUA, and EAU).
5 experts selected the most appropriate questions for the final draft (EF, MCM, CV, PM, MTM)
3 authors (EF, MCM, MTM) prepared the final manuscript design
Screening
131 questions about the robotic approach to partial nephrectomy were selected as a final questionnaire
The questions were organized in topics and sent by email to all authors. Only one round of questions was performed
Eligibility
We considered as consensus all questions with more than 75% concordance
Included
After the authors' approval, each question's content was included in the final manuscript as a subtopic in the article, and we described the expert's opinions on each subject
